# Amperometric Biosensor Based on Diamine Oxidase/Platinum Nanoparticles/Graphene/Chitosan Modified Screen-Printed Carbon Electrode for Histamine Detection

**DOI:** 10.3390/s16040422

**Published:** 2016-03-24

**Authors:** Irina Mirela Apetrei, Constantin Apetrei

**Affiliations:** 1 Department of Pharmaceutical Sciences, Faculty of Medicine and Pharmacy, “Dunarea de Jos” University of Galati, 47 Domneasca Street, 800008 Galati, Romania; irina.apetrei@ugal.ro; 2 Department of Chemistry, Physics and Environment, Faculty of Sciences and Environment, “Dunarea de Jos” University of Galati, 47 Domneasca Street, 800008 Galati, Romania

**Keywords:** diamine oxidase, graphene, biosensor, histamine, fish

## Abstract

This work describes the development and optimization studies of a novel biosensor employed in the detection and quantification of histamine in freshwater fish samples. The proposed biosensor is based on a modified carbon screen-printed electrode with diamineoxidase, graphene and platinum nanoparticles, which detects the hydrogen peroxide formed by the chemical process biocatalysed by the enzyme diamine oxidase and immobilized onto the nanostructurated surface of the receptor element. The amperometric measurements with the biosensor have been implemented in buffer solution of pH 7.4, applying an optimal low potential of +0.4 V. The novel biosensor shows high sensitivity (0.0631 μA·μM), low detection limit (2.54 × 10^−8^ M) and a broad linear domain from 0.1 to 300 μM. The applicability in natural complex samples and the analytical parameters of this enzyme sensor have been performed in the quantification of histamine in freshwater fish. An excellent correlation among results achieved with the developed biosensor and results found with the standard method for all freshwater fish samples has been achieved.

## 1. Introduction

Biogenic amines are compounds of an organic nature with relatively low molecular weight and basic character, with different chemical structures containing at least one nitrogen atom, formed mainly by the decarboxylation process of alpha amino acids resulting from the hydrolysis of proteins [1].

Biogenic amines are usually recognized as key compounds, indicating the microbiologic decay in fish, fish products or shellfish [2,3]. Biogenic amines (BAs) are even found in different foods [4,5,6] and beverages [7,8] and could also be found in meat, fermented foods, cheese or wine [1].

The major biogenic amines (BAs) as quantity and biological or nutritional implication and also related to spoilage in meat and fish are putrescine (1,4-diaminobutane), cadaverine (1,5-diaminopentane), tyramine (2-(*p*-hydroxyphenyl)ethylamine) and histamine (2-(4-imidazolyl)ethylamine). However, histamine is one of the most biochemically active compounds from this class of compounds [9]. Consequently, it is essential to find out the amount of histamine in samples because it provokes scombroid syndrome, even though altering of the aspect, color and odor of the fish are not observed [10]. When ingested, these compounds have a negative effect on the heart’s normal functions, motor neurons, smooth muscle and stomach [10].

Histamine appears in fresh fish as a consequence of unsuitable manipulation, refrigeration or conservation after catching [11]. Therefore, it is essential to implement fast, sensitive, selective and economical methods for the detection and quantification of histamine. These methods could be alternative or complementary to the classical methods, which principally consist of chromatography, for instance high performance liquid chromatography [12], cation-exchange chromatography [13], gas chromatography [14] or thin layer chromatography [15]. Detection of BAs by chromatographic methods requires derivation stage, pre- or post-column, to improve BA detection in ultraviolet, increasing the time of analysis and its cost. Other detection methods such as spectrophotometric methods [16,17], fluorometric methods [18] or chemiluminometric methods [19] have also been applied. For the quantification of BAs, biosensors could be attractive analytical tools offering fast and reduced time analysis methods, adequate selectivity and sensitivity, and low cost [20,21]. Furthermore, the biosensors could be used for rapid screening of aminic compounds [22,23].

Graphene has newly become an appealing nanomaterial in a multitude of applications due to its excellent physical, mechanical, electronic and chemical properties [24,25]. In the last few years, graphene (GPH) has been used as a nanostructured modifier material of working electrodes for development of electrochemical sensors and biosensors, gas sensors, fuel cell, *etc.* [26,27,28].

Platinum or gold nanoparticles reveal catalytic effects on hydrogen peroxide (H_2_O_2_) electrochemistry and were extensively used in the field of sensors in different applications [29,30]. It could be of large interest to develop platinum nanoparticles (nPt)—graphene (GPH) nanocomposites as sensitive layers of novel biosensors. The synergy between electrocatalytic properties and the high rate of electron transfer functionalized GPH and platinum nanoparticles may significantly improve characteristics of the biosensor.

In this paper, we present a novel biosensor for the accurate quantification of histamine. The sensitive element is based on diamine oxidase immobilized into a nanostructured nPt/GPH/chitosan thick film of a modified carbon screen-printed electrode. For this purpose, several experimental parameters have been studied with the aim of identifying the most favorable ones that improve its electrochemical signal characteristics. In addition, the novel biosensor has been validated in the laboratory regarding the quantification of the histamine amount in several freshwater fish samples to evaluate the applicability in practice. An excellent correlation between the histamine amount values obtained with the biosensor and the histamine amount values obtained by ELISA (enzyme-linked immunosorbent assay ) has been established.

## 2. Materials and Methods

### 2.1. Chemicals

Diamine oxidase (from porcine kidney, ≥0.05 unit·mg^−1^ solid, EC 1.4.3.6, DAO), histamine, chitosan, acetic acid, phosphate buffer solutions, potassium chloride, sulphuric acid, potassium tetrachloroplatinate(II), hydrogen peroxide (H_2_O_2_), l-lysine (Lys), l-tyrosine (Tyr), l-histidine (His), l-tryptophan (Trp), putrescine (1,4-diaminobutane), cadaverine (1,5-diaminopentane), and tyramine (2-(*p*-Hydroxyphenyl)ethylamine) were all acquired from Sigma-Aldrich (St. Louis, MO, USA). The Veratox^®^ kit ELISA, dedicated to histamine quantification, was purchased from Neogen^®^ Corporation (Lansing, MI, USA). All aqueous solutions required in the electrochemical studies were freshly prepared from the solid and pure compounds with ultrapure water (resistivity 18.3 MΩ·cm) obtained in the lab with professional equipment (Milli-Q Simplicity^®^ Water Purification System from Millipore Corporation, Billerica, MA, USA).

### 2.2. Materials

Reduced graphene oxide (GPH) from Sigma-Aldrich (St. Louis, MO, USA) was used for carbon screen printed electrode (CSPE) modification. Carbon screen-printed electrodes, model 110 working in solution (with working electrodes of 4 mm in diameter) were acquired from Dropsens Ltd. (Llanera, Spain). CSPEs were modified with graphene, chitosan, Pt nanoparticles and DAO in order to obtain a biosensor.

### 2.3. Development of GPH/Chitosan/CSPE

The GPH dispersion was prepared from 1 mg GPH dispersed in 1 mL chitosan solution (0.2% in acetic acid, pH 5). The heterogeneous system was ultrasonicated for 2 h in order to achieve a homogeneous dispersion of GPH in the liquid phase. GPH-modified carbon screen-printed electrodes were prepared by the drop-and-dry method. In addition, 10 mL of 1 mg·mL^−1^ GPH dispersion were cast on the CSPE (diameter of 4 mm) and left to dry slowly at room temperature in a desiccant.

### 2.4. Development of Pt Nanoparticles/GPH/Chitosan/CSPE

Platinum nanoparticles (nPt) were deposited on the GPH/chitosan/CSPE applying a potential of +0.2 V (referred to the standard hydrogen electrode) for an established period of time from a 2 × 10^−3^ M H_2_PtCl_4_ (Potassium tetrachloroplatinate(II) + sulphuric acid) solution. Solution employed in the electrosynthesis of Pt nanoparticles was deoxygenated by purging nitrogen. In the electrodeposition process, a Pt gauze (1 cm^2^) and Ag/AgCl electrode were employed as auxiliary (counter) and reference electrodes, respectively. After the deposition of Pt nanoparticles, the modified electrode was carefully rinsed with ultrapure water. The modified electrodes were left to dry in a desiccator at room temperature overnight [31].

### 2.5. Development of DAO-nPt/GPH/Chitosan/CSPE

For the experiment, 10 mg·mL^−1^ solution of DAO was prepared using 0.2% chitosan solution as a solvent. Next, 10 mL of DAO solution were added on the surface of nPt/GPH/chitosan/CSPE. Immediately after drying, the nPt/GPH/chitosan/CSPE modified with DAO was kept at 4 °C to dry during 12 h. After the drying step, the DAO-nPt/GPH/chitosan/CSPEs were kept in a desiccator at 4 °C. Degradation of enzymes is reduced in these conditions of storage.

### 2.6. Apparatus

Cyclic voltammetry and amperometry techniques were carried out using a Biologic SP 150 potentiostat/galvanostat (Bio-Logic Science Instruments SAS, Claix, France). The control and data acquisition were carried out using EC-Lab Express software (A three-electrode cell of 25 mL capacity was employed for the electrochemical experiments. The connection to potentiostat was assured by a special cable permitting the use of all electrodes from the commercial screen-printed electrode device. The biosensor (DAO-nPt/GPH/chitosan/CSPE) was the working electrode. Pseudo-reference electrode of Ag and auxiliary (counter) electrode of C completed the electrochemical system. The amperometric signals of biosensor were registered applying +0.4 V in a uniformly stirred solution. Supporting electrolytes were phosphate buffer solution (PBS) (pH = 7.4).

The buffer solutions with different pHs were prepared and the pHs were evaluated with a pH-meter (Inolab pH 7310). For the freshwater fish sample pre-treatment, a centrifuge was necessary (Cencom II).

### 2.7. ELISA Measurements

Histamine contents of freshwater fish samples were analyzed with a standard method to validate the measurements with the biosensor. The standard method employed for quantification of histamine in freshwater fish samples was the ELISA method based on Neogen’s Veratox kit (Lansing, MI, USA). This kit could be used for detection of the histamine amount ranging from 2.5 to 40 ppm. The analysis principle is one competitive direct ELISA. Histamine from the real sample and control samples compete with enzyme-labeled histamine for the antibody-binding sites. After a wash stage, substrate reacted with the bound enzyme conjugate. The reaction generated a color, from blue to red. A microwell reader was employed to obtain the optical densities at 620 nm. Optical densities of control samples were used to develop the calibration curve. Optical densities of samples are interpolation in the calibration curve to calculate concentration of histamine in each sample.

### 2.8. Freshwater Fish Samples

Different freshwater fish samples including carp (*Cyprinus carpio*), Prussian carp (*Carassius gibelio*), tench (*Tinca tinca*), Wels catfish (*Silurus glanis*) and European perch (*Perca fluviatilis*) were purchased from a traditional fish market. The samples were analyzed with the aim of testing the performance characteristics of the novel biosensor. The fish samples were analyzed in two freshness stages, immediately after acquisition and after 48 h. Previous to performing the ELISA and amperometric determinations, fish samples were pre-processed. Biogenic amines were extracted in an aqueous liquid phase useful for electrochemical analysis. For this purpose, fish samples were cleaned, eviscerated, washed and then cut in thick slices. The slices were blended, obtaining a homogenous paste. Five grams of this paste were mixed with 45 mL of ultrapure water. Diluted paste was introduced in an extraction funnel and shaken vigorously for 10 min. It assured an efficient extraction process. After the separation of liquid phase, the same process was repeated twice. Finally, the separated liquid phase was centrifuged at 4000 rot/min for five minutes. The supernatant was the sample used in electrochemical analysis with the biosensor [32].

## 3. Results and Discussion

### 3.1. Exploratory Studies by Cyclic Voltammetry

**Figure 1:**
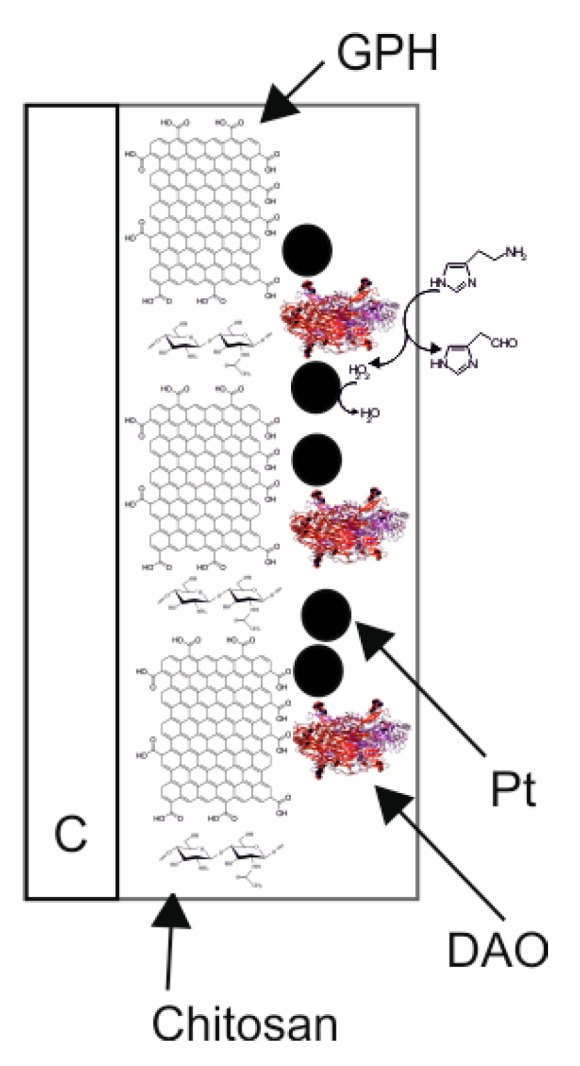
Scheme of the sensitive layer and the enzymatic/electrochemical processes of biosensor.

In the present work, the electrochemical signal of the DAO-nPt/GPH/chitosan/CSPE biosensor is principally associated with the oxidation process of hydrogen peroxide (H_2_O_2_), which is the enzymatic product of interaction between DAO and histamine. Components of the sensitive layer and the scheme of the electrochemical and enzymatic/electrochemical processes at the level of biosensor active surface are depicted in Figure 1.

**Scheme 1:**
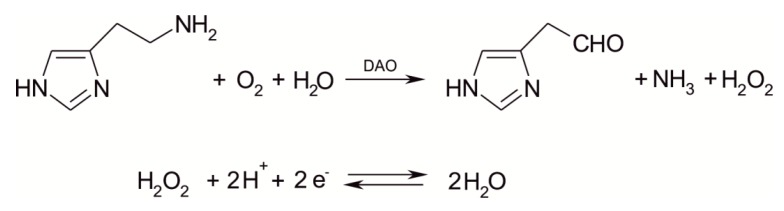
Enzymatic and electrochemical processes at the level of detector element of the biosensor.

The enzymatic and electrochemical processes at the level of detector element of the biosensor are presented in the following Scheme 1.

It is well-known that H_2_O_2_ could be electrochemically detected by oxidation employing solid metal based electrodes [33]. Usually, the redox process of H_2_O_2_ takes place at high potential values causing possible interference from other compounds with redox properties present in samples. Noble metals nanoparticles, such as platinum or gold, were extensively used in electrochemistry as catalysts for the redox process of H_2_O_2_ [34]. In order to demonstrate the electrocatalytic properties of the GPH/chitosan and nPt/GPH/chitosan nanostructured layers, used for modification of electrodes, to H_2_O_2_ oxidation, cyclic voltammograms were registered. The electrochemical signals of all electrodes do not present any electrochemical peaks in PBS of pH = 7.4 if the sample does not contain H_2_O_2_ (data not shown).

**Figure 2:**
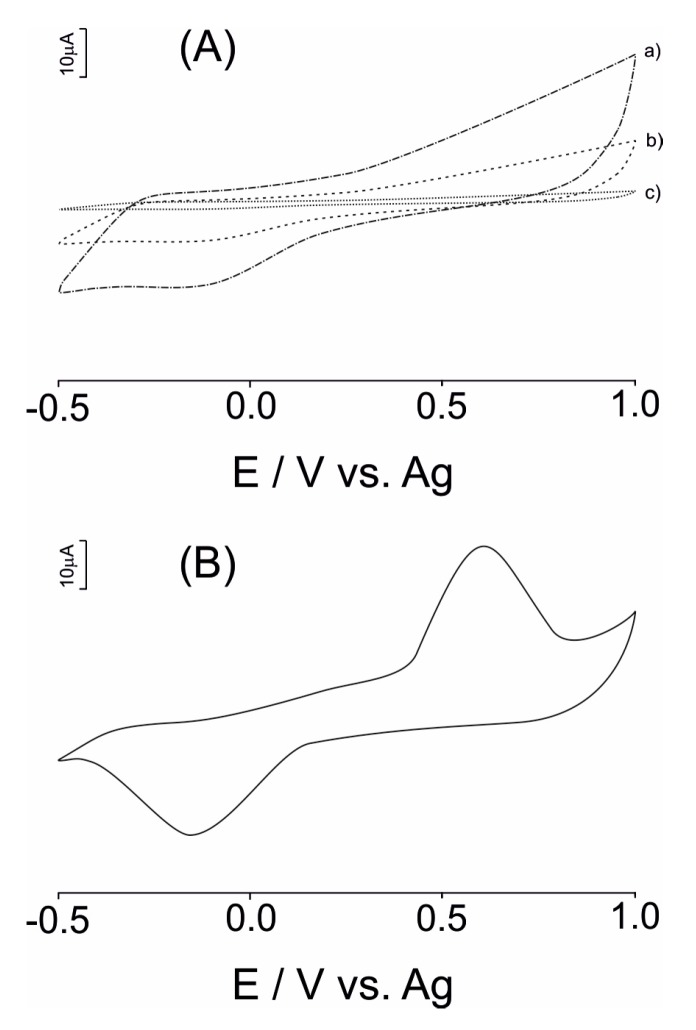
(**A**) CVs registered at (**a**) CSPE; (**b**) GPH/chitosan/CSPE; (**c**) nPt/GPH/chitosan nanocomposite film in 10^−4^ M H_2_O_2_ solution (supporting electrolyte 10^−2^ M PBS of pH = 7.4); (**B**) cyclic voltammogram of the DAO-nPt/GPH/chitosan/CSPE biosensor in a 10^−2^ M PBS of pH = 7.4 containing 10^−4^ M histamine.

Figure 2A shows the cyclic voltammograms (CV) of modified SPE with an nPt/GPH/chitosan nanostructured thick film in a 10^−4^ M H_2_O_2_ solution (supporting electrolyte 10^−2^ M PBS of pH = 7.4) (curve a). In the same figure, the CVs of GPH/chitosan/CSPE (curve b) and unmodified CSPEs (curve c) are shown.

As observed in Figure 2, the redox peak currents related to H_2_O_2_ at the CSPE are very small, and the oxidation process of H_2_O_2_ starts at 0.55 V. In contrast, the electrochemical currents observed at the GPH/chitosan/CSPE and the nPt/GPH/chitosan/CSPE are greater than the currents observed at the CSPE. In addition, the oxidation process of H_2_O_2_ started at a relative low potential of 0.25 V. Electrochemical reduction process of H_2_O_2_ started at a potential of 0.15 V for modified electrodes. Furthermore, the reduction (cathodic) current related to H_2_O_2_ increased when the potential applied decreased.

The important decrease of the redox potential of H_2_O_2_ is assigned to the electrocatalytic properties of the nPt and GPH. Moreover, the magnitude (peak highness) of the oxido-reduction currents increased in the subsequent order for the modified electrodes developed in this work: nPt/GPH/chitosan/CSPE > GPH/chitosan/CSPE > CSPE. From these results, the synergistic effect of GPH and nPt in nanocomposite film on the electrochemical detection of H_2_O_2_ was demonstrated. From all electrodes, the nPt/GPH/chitosan/CSPE revealed the highest sensitivity towards H_2_O_2_.

The influence of time necessary for electrochemical deposition of nPt on the sensitivity of nPt/GPH/chitosan/CSPE towards H_2_O_2_ was studied. The sensitivity of nPt/GPH/chitosan/CSPE was studied by chronoamperometry applying +0.4 V in 10^−3^ M H_2_O_2_ aqueous solution. Both oxidation and reduction currents increased with the increment of deposition time. After 300 s of deposition time, the oxidation and reduction currents reach a plateau. If the deposition time is greater than 300 s, the electrochemical signal of electrode starts decreasing, which could be related to the reduction of electroactive surface area. At greater deposition time, microparticles were deposited instead of nanoparticles, decreasing the number of active centers. Therefore, 300 s is optimal for the deposition of nPt for a major sensitivity.

Figure 2B shows the CV of the DAO-nPt/GPH/chitosan/CSPE biosensor in a 10^−2^ M PBS of pH = 7.4 containing 10^−4^ M histamine. In the cyclic voltammogram of the DAO-nPt/GPH/chitosan/CSPE in 10^−5^ M histamine solution, an anodic wave related to the oxidation process of H_2_O_2_ at +0.60 V and a cathodic peak at −0.15 V related to the reduction process of H_2_O_2_ is observed, respectively. Diamine oxidase catalyzes the oxidation process of histamine. From the reaction products, H_2_O_2_ is electrochemically detected at biosensor surface at a low potential value. This is the detection principle of the biosensor based on DAO, nPt and GPH developed in this study.

The effect of the scan rate in the biosensor electrochemical response was studied by registering the CVs of the DAO-nPt/GPH/chitosan/CSPE biosensor at different sweep rates, in the range 0.05–1.00 V·s^−1^. The intensity of peak associated with the oxidation process of H_2_O_2_ follows the linear dependence described by the equation, I = 9 × 10^−5^ × v^1/2^ + 7 × 10^−6^ proving that the process is one controlled by diffusion, in agreement with the Randles–Sevcik equation [35]:

I_pa_ = 2.687 × 10^5^ × n^3/2^ × ν^1/2^ × D^1/2^ × A × C (1)

where I_pa_, n, ν, D, A and C have their usual meanings [4]. From the linear adjustment between I_pa_ and ν^1/2^ (the slope), the electrode surface area, A, was calculated. Diffusion coefficient of H_2_O_2_ is 6 × 10^−6^ cm^2^·s^−1^ [36]. The calculated electrode surface area was 1.2434 ± 0.0121 cm^2^. The electroactive area of the biosensor is much larger than the geometrical electrode surface area (0.1256 cm^2^). Therefore, the DAO-nPt/GPH/chitosan/CSPE biosensor presents fast electrochemical processes, increasing sensitivity of the biosensor.

### 3.2. Optimization of Working Conditions

For setting up the biosensor, for the optimal applied potential in order to achieve the highest sensitive detection of biogenic amine histamine, the electrochemical responses (current) were measured, varying the potential applied to biosensor. The presence of –COOH and –OH groups facilitates the immobilization of DAO on the biosensor surface (nPt/GPH/chitosan) by means of electrostatic, hydrophobic, van der Waals, hydrogen bonding interactions, and a combination of those [37]. The sensitive layer of biosensor is stable and the cross-linking process is not necessary, resulting in an increasing of the biosensor sensitivity [38].

As highlighted before, the biosensor amperometric response is related to the H_2_O_2_ resulting in the biocatalytic process, which usually occurs at a high potential values. At the level of this novel biosensor, the enzymatic system synergetic with GPH, increases the electroactive surface of biosensor and facilitates the electron transfer and nPt as a catalyst for oxido-reduction of H_2_O_2_ facilitates, decreasing this oxido-reduction potential to +0.4 V *vs.* Ag.

**Figure 3:**
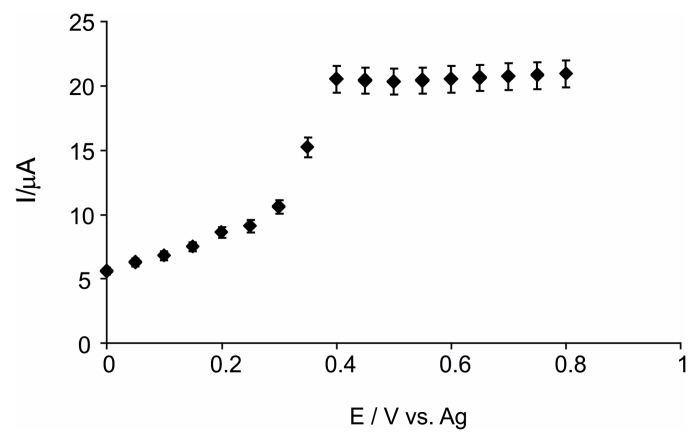
Current-potential dependence.

The amperometric response of the biosensor was determined under continuous stirring of the histamine solution in order to obtain the optimal value of applied potential. The optimal applied potential is one important parameter that decisively influences the biosensor electrochemical signal to histamine, for both selectivity and sensitivity. Potential range analyzed was among 0.0 V *vs.* Ag and 0.8 V *vs.* Ag. The most intense response is obtained at +0.4 V *vs.* Ag. Therefore, a potential value of +0.4 V *vs.* Ag was selected because, at this value, the highest response of biosensor to histamine (10^−4^ M) is achieved, *versus* the response of biosensor in blank electrolyte solution (PBS). (Figure 3).

pH is another very important factor influencing the enzymatic activity. Therefore, the effect of pH in the DAO-nPt/GPH/chitosan/CSPE signal was studied varying the PBS pHs (it was used as support electrolyte). The effect of pH in the DAO-nPt/GPH/chitosan/CSPE response detecting 10^−4^ M histamine in the range from 6.4 to 8.4 was investigated.

**Figure 4:**
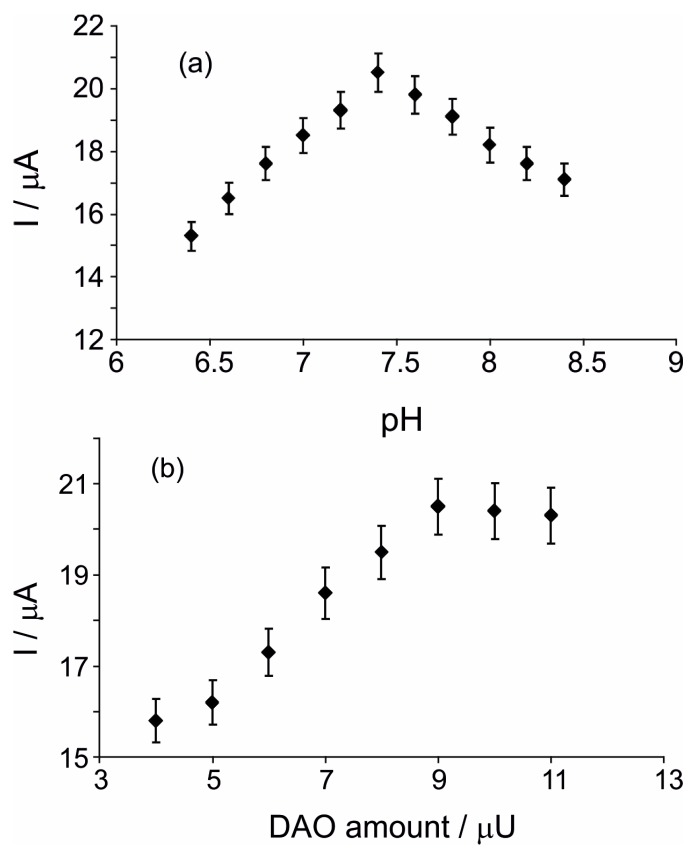
(**a**) Effect of the pH in detection of 10^−4^ M histamine in 10^−2^ M PBSs with different pHs. Electrochemical experiments were carried out at +0.4 V in triplicate (RSD = 2.24%); (**b**) Variation of biosensor response *versus* the amount of DAO immobilized on surface. Measurements were performed at +0.4 V in triplicate (RSD = 4.46%) in 10^−4^ M histamine solution (electrolyte support 10^−2^ M PBS of pH = 7.4).

As shown in Figure 4a, the biosensor response increased with the increasing of pH, achieving a maximum at pH 7.4. Greater values, in the range from 7.4 to 8.4 caused a decreasing of the biosensor response. Consequently, phosphate buffer solution with pH 7.4 was used for the electrochemical measurements with the biosensor.

In order to find the optimal quantity of diamineoxidase on the surface of nPt/GPH/chitosan/CSPE, various biosensors were developed, increasing the quantity of DAO immobilized. Figure 4b illustrates the variation of biosensor response *versus* the amount of DAO immobilized on the surface. The peak current increases when the DAO quantity is increased. If the quantity is greater than 9 μU of DAO, a slight diminishing of the biosensor response could be observed. This tendency points out that, at relatively small quantities, DAO immobilized increases the rate of the histamine enzymatic process. High quantities of DAO immobilized might cause a limitation of histamine diffusion to the active center of DAO. Consequently, 9 μU of DAO was employed to develop the optimal biosensor, because no considerable differences on the biosensor response among 9 μU and 11 μU of DAO were noticed.

### 3.3. Characteristics of the DAO/nPt/GPH/Chitosan/CSPE Biosensor

The histamine biosensor was further characterized regarding the principal analytical performance characteristics, such as linear interval concentration range, sensitivity and detection limit, interferences, reproducibility, operational and storage stability.

**Figure 5:**
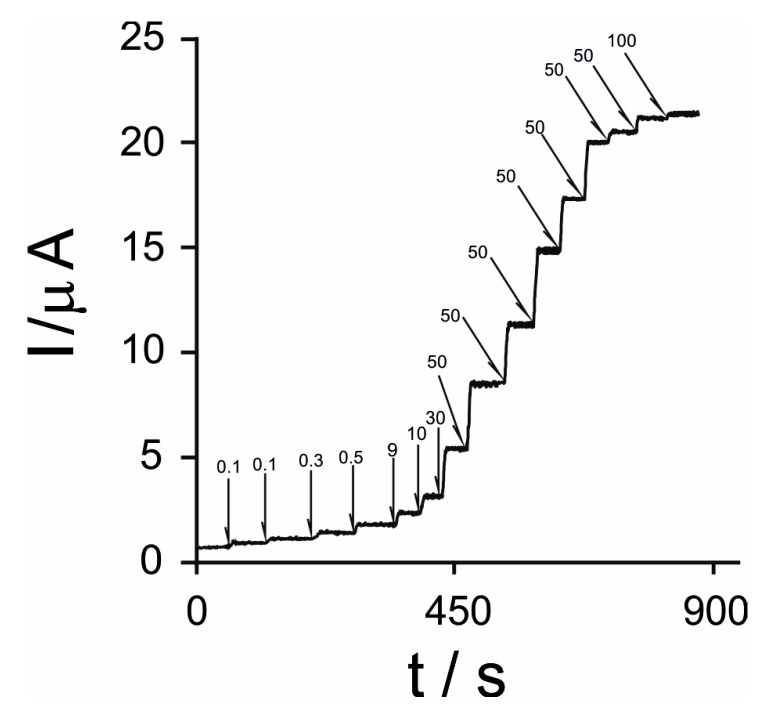
Amperometric signal of DAO/nPt/GPH/chitosan/CSPE to consecutive additions of histamine in a concentration range from 0.1 μM to 100 μM at +0.4 V. The values from the figure are expressed in μM for each step.

The chronoamperometric technique was employed for the characterization of the diamine oxidase/platinum nanoparticles/grapheme-biosensor detecting histamine. Figure 5 shows the usual amperometric response of DAO/nPt/GPH/chitosan/CSPE to consecutive additions of histamine applying a potential of +0.4 V.

As can be noticed in Figure 5, the amperometric signal of the biosensor increased if the histamine concentration augmented.

**Figure 6:**
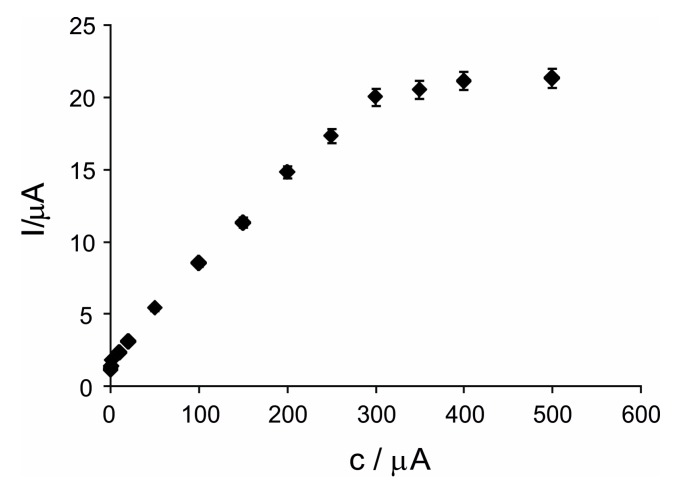
The calibration curve of biosensor detecting histamine.

Figure 6 shows the calibration plot of the anodic current *vs.* the concentration of histamine detected by biosensor.

As can be noticed in Figure 6, this novel biosensor has a relatively broad linearity range, from 0.1 μM to 300 μM of histamine. The biosensor’s linear equation is I = 0.0631 × c + 1.6633 with a coefficient of determination of 0.9962. The detection limit (LOD) of the histamine biosensor is 2.54 × 10^−8^ M calculated by means of 3 × σ/m criteria (where σ is standard deviation and m is slope (sensitivity) of the calibration plot). Enhanced sensitivity of this novel biosensor (0.0631 μA·μM, R^2^ = 0.9962) could be related to a considerable surface area of the electrode, to the fast electrons transfer facilitated by GPH from the immobilization matrix, and to the catalytic synergism of nPt and GPH in the electrochemical processes of H_2_O_2_. Inside of the nanocomposite sensitive layer, DAO is contacting both GPH and nPt. This synergism makes possible one rapid electron exchange between the DAO and the CSPE with a small energetic barrier. The histamine biosensor, DAO/nPt/GPH/chitosan/CSPE, reaches a superior LOD compared with the DAO/graphite with peroxidase and Osmium mediator biosensor [39] (a detection limit of 5 μM), DAO/hydrogel film of photo-2-hydroxyethyl methacrylate/carbon paste SPE biosensor [40] (a detection limit of 0.65 ppm), DAO-HRP/C-SPE biosensor [41] (a detection limit of 0.40 μM), and DAO/Pt/Copolymerization (GA-membrane) biosensor [42] (a detection limit of 25 μM).

Hill coefficient (h) was estimated from the current-concentration dependence by plotting log[I/(I_max_ − I)] *vs.* log [histamine] (molar concentration of histamine). Hill coefficient value calculated was 1.02 ± 0.02. This value close to 1 (ideal value) demonstrates that the enzymatic process of histamine at the biosensor surface follows a typical Michaelis–Menten kinetics [35].

The parameters of Michaelis-Menten kinetics were calculated. The Lineweaver-Burk equation adapted for biosensors is presented below [43]:

(2) $$\frac{1}{I}=\frac{1}{I_{\max}}+\frac{K_{M}^{app}}{I_{\max}\left[S_{ox}\right]}$$

where $K_{M}^{app}$—apparent Michaelis-Menten constant; [S]—molar concentration of histamine; I—biosensor response current; I_max_—steady-state current.

The I_max_ value calculated is 22.4 μA and $K_{M}^{app}$ = 120.6 μM. The $K_{M}^{app}$ obtained for this novel biosensor is lower but comparable with $K_{M}^{app}$ obtained for other biosensors detecting histamine [42]. These Michaelis-Menten parameters calculated demonstrate an excellent sensitivity (low $K_{M}^{app}$ and high I_max_) of the biosensor based on DAO, nPt and GPH for the detection and quantification of histamine.

### 3.4. Biosensor Stability Studies

Relative standard deviation of the biosensor electrochemical signal towards 10^−4^ M histamine was 5.5% for five different biosensors fabricated in identical conditions. These results demonstrate an excellent reproducibility of the fabrication process.

**Figure 7:**
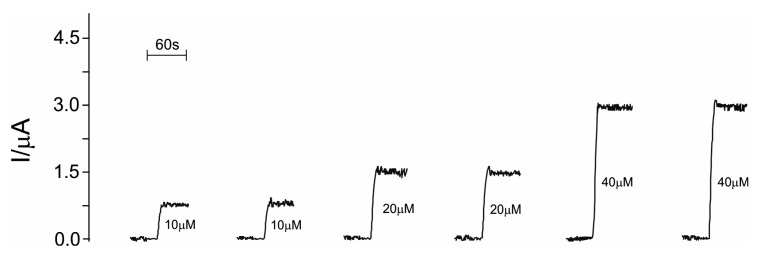
Responses of biosensor immersed in solutions of histamine with different concentrations (10 μM, 20 μM and 40 μM) with an intermediary step of biosensor rinsing. Applied potential +0.4 V; supporting electrolyte 10^−2^ M PBS pH = 7.4.

In Figure 7, the measurements with the biosensor by changing concentrations of histamine with a rinsing step with PBS between subsequent measurements is shown.

As can be observed, the biosensor reaches the baseline after each measurement, indicating the viability of biosensor in the detection of histamine.

Storage stability of DAO/nPt/GPH/chitosan/CSPE was studied following the procedure described below. Two biosensors were fabricated in an identical mode and their sensitivity toward 10^−4^ M histamine was determined over 30 days. The amperometric response of one biosensor was registered in triplicate in each day for a period of 30 days (one month). The response of the second biosensor was determined just after its fabrication and on the 30th day. During this time, DAO/nPt/GPH/chitosan/CSPE were stored in a desiccator at 4 °C. The results obtained showed adequate and comparable stability during storage in both conditions, because the decrease of the biosensor response was only 10.2 and 12.6%, respectively, after one month.

**Figure 8:**
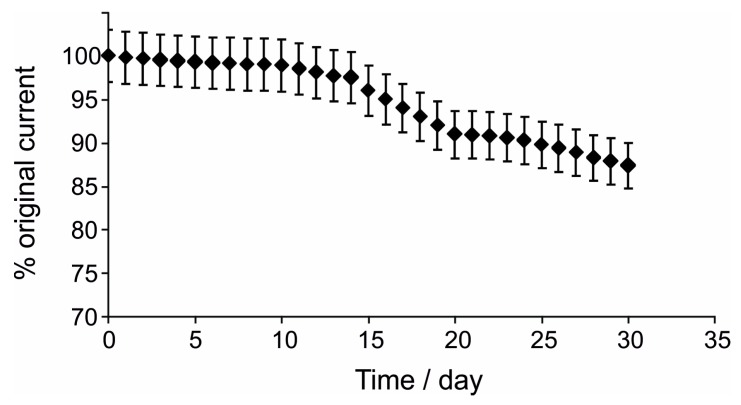
Operational stability of the biosensor.

A plot of the results of operational stability measurements over time is depicted in Figure 8.

As seen in Figure 8, for the daily use of the biosensor, a very slow decrease in the biosensor amperometric response was observed in the first 10 days (1.1%). Subsequently, the biosensor response decreased gradually, more accentuated, reaching 12.6% after 30 days.

### 3.5. Interferences in Biosensor Response

The interfering study is an essential stage before real sample analyses. For this purpose, the influence of several amino acid precursors of the principal biogenic amines such as His, Tyr, Lys and Trp as well as biogenic amines such as putrescine, cadaverine and tyramine in biosensor response was studied. The influence of these compounds was examined, evaluating the amperometric responses obtained for two standard solutions of histamine (10^−5^ M and a 5 × 10^−6^ M), and the amperometric responses of the same solutions contain the same concentration of interfering amino acids or other biogenic amines.

**Figure 9:**
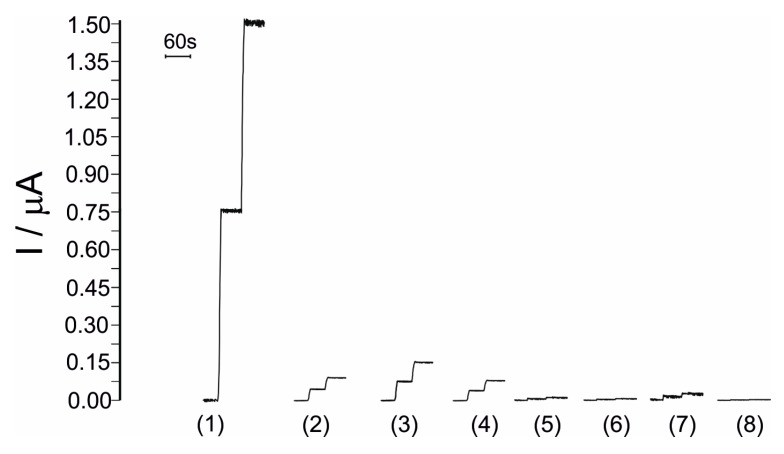
Comparison between the amperometric responses of biosensor towards (**1**) histamine; (**2**) cadaverine; (**3**) tyramine; (**4**) putrescine; (**5**) Tyr; (**6**) Lys; (**7**) Trp; (**8**) His.

In Figure 9, the responses of biosensors towards histamine 10^−5^ M and interfering compounds 10^−5^ M in the optimal conditions are presented.

Results obtained reveal an insignificant influence of amino acids in the amperometric detection and quantification of histamine, in the error limit range (3%). Influence of other biogenic amines in the amperometric detection and quantification of histamine is greater than those observed for amino acids but lower than 10% in all cases.

### 3.6. Real Sample Analysis: Application in Freshwater Fish Samples

Different freshwater fish samples were analyzed with the DAO/nPt/GPH/chitosan/CSPE biosensor for histamine detection and quantification. The purpose of this relatively high diversity of samples was to evaluate if the quantification of the histamine amount in different freshwater fish species could be carried out with good reliability.

Histamine quantification was performed by interpolation of biosensor response in the calibration plot. For the recovery studies and for the influence of the matrix effect in the detection of histamine, the standard addition procedure was implemented. In the optimal experimental conditions, amperometric measurements with biosensors were performed in triplicate. The results are presented in Table 1.

As can be noticed in Table 1, there are relatively small differences between direct interpolation and addition methods. Therefore, the matrix interfering effect is not significant in histamine detection in freshwater fish samples.

**Figure 10:**
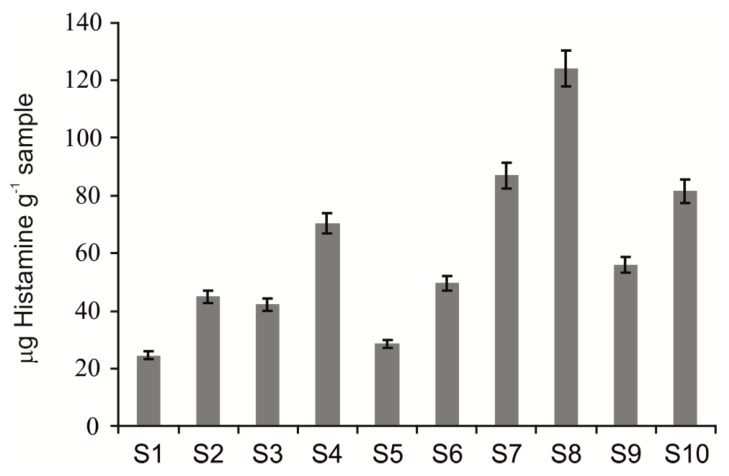
Increments of total histamine amount in freshwater fish samples. Values reported are the average of all quantification methods. S1—Carp (fresh); S2—Carp (48 h); S3—Tench (fresh); S4—Tench (48 h); S5—Prussian carp (fresh); S6—Prussian carp (48 h); S7—European perch (fresh); S8—European perch (48 h); S9—Wels catfish (fresh); S10—Wels catfish (48 h).

The freshness evolution was followed by the quantification of histamine amount in all freshwater fish samples stored at 4 °C. Histamine contents were determined initially (very fresh samples) and after 48 h of storage for all fish samples. The results obtained are revealed in Figure 10 in the form of a bar diagram.

For all samples in the study, the histamine contents increased after 48 h of storage. It is recognized that histamine amount levels could be considered specific and reliable indicators of fish freshness. The quality of fish is also related to histamine content. Quality control and freshness are important for fish samples in order to prevent scombroid syndrome, which is related to the ingestion of spoiled fish. It could be successfully implemented with this novel biosensor.

As a supplementary confirmation of the method based on a DAO-nPt/GPH/chitosan/CSPE biosensor, was based on a Student’s paired samples *t*-test (95% confidence level, nine degrees of freedom). The Student’s *t*-test compares the results of the biosensor method with the results of the ELISA method. When comparing the results found by the interpolation method and the standard addition method, the experimental *t*-value obtained was 0.4678, whereas the critical tabulated *t*-value was 2.2621. Therefore, the differences between the histamine concentrations obtained by the interpolation method and the standard addition method are not statistically significant. In addition, experimentally obtained *t*-values were 0.1678 and 0.7263, respectively, when the interpolation method *vs.* ELISA and the standard addition method *vs.* ELISA were compared. Experimental *t*-values are lower compared with the theoretical critical *t*-value of 2.2621. Therefore, the differences between histamine concentrations obtained by means of the biosensor and the ELISA are not statistically significant, confirming the feasibility of the biosensor.

## 4. Conclusions

We developed a novel nanobiocomposite thick film of DAO/nPt/GPH/chitosan for histamine biosensing. The biosensor reveals excellent sensitivity to histamine (0.0631 μA·μM) and a low detection limit (2.54 × 10^−8^ M). Enhanced sensitivity is related to the electrocatalytic synergetic effect of GPH and nPt on the electrochemical detection of H_2_O_2_. A DAO/nPt/GPH/chitosan based biosensor has a sensitive amperometric response towards histamine, which could be related to the huge electroactive area and to the rapid electron exchange mediated by nPt and GPH. The biosensor fabrication is highly reproducible and the biosensor presents excellent stability. l-hystidine, l-tyrosine, l-lysine and l-tryptophan interferences are insignificant in the biosensor electrochemical response detecting histamine. Interference of biogenic amines is reduced. The histamine content in freshwater fish samples was reliably quantified with the biosensor. Histamine amounts in fish samples were obtained by the interpolation method and standard addition method with good precision. Excellent correlations between the histamine amounts, obtained with the developed biosensor and ELISA method, were found.

## Figures and Tables

**Table 1:** Quantities of histamine in freshwater fish samples under study. Data are based on the biosensor method and ELISA method, respectively.

Sample	μg Histamine·g^−1^ Sample		
	Interpolation ^1^	Standard Addition ^1^	ELISA
Carp (fresh)	24 ± 2	25± 2	25
Carp (48 h)	44 ± 4	46 ± 4	45
Tench (fresh)	43 ± 4	42 ± 5	42
Tench (48 h)	71 ± 6	70 ± 6	70
Prussian carp (fresh)	28 ± 2	29 ± 2	29
Prussian carp (48 h)	50± 4	49± 4	50
European perch (fresh)	86 ± 8	88 ± 8	87
European perch (48 h)	124± 9	123± 9	125
Wels catfish (fresh)	56 ± 6	56 ± 6	56
Wels catfish (48 h)	81 ± 8	82 ± 8	82

^1^ *n* = 3; 95% confidence level.
